# Contemporary Transcatheter Approaches to Mitral Regurgitation

**DOI:** 10.31083/RCM49455

**Published:** 2026-05-26

**Authors:** Panagiotis Theofilis, Panagiotis Iliakis, Kyriakos Dimitriadis, Paschalis Karakasis, Panayotis K. Vlachakis, Eirini Dri, Konstantinos Pamporis, Alexios Antonopoulos, Constantina Aggeli, Konstantinos Aznaouridis, Dimitris Tousoulis, Konstantinos Tsioufis

**Affiliations:** ^1^Department of Cardiology, “Hippokration” General Hospital of Athens, 11527 Athens, Greece; ^2^Department of Cardiology, Ippokrateio General Hospital of Thessaloniki, 54642 Thessaloniki, Greece

**Keywords:** mitral regurgitation, transcatheter mitral valve repair, MitraClip, PASCAL, annuloplasty, transcatheter mitral valve replacement

## Abstract

Mitral regurgitation (MR) is a prevalent and prognostically relevant valvular disease, especially in patients with heart failure, in whom MR contributes to adverse remodeling, increased symptom burden, and higher mortality. Surgical repair or replacement remains the standard of care for suitable candidates, but many patients are excluded because of advanced age, comorbidities, or high surgical risk. Transcatheter methods have emerged as transformative alternatives, including mitral transcatheter edge-to-edge repair (MTEER) with devices such as MitraClip and PASCAL, annuloplasty-based devices such as Carillon and Cardioband, and transcatheter mitral valve replacement (TMVR) with devices such as Tendyne, Intrepid, and others under development. Data from randomized trials and registries have established that MTEER lowers hospital readmission rates and improves mortality in carefully selected secondary MR subjects, and that device upgrades improve procedural success and anatomical versatility. Annuloplasty provides targeted repair for functional MR with annular dilation, whereas TMVR offers an alternative for anatomically complex cases or those ineligible for MTEER, albeit with distinct procedural risks. Management of severe mitral annular calcification remains difficult and demands meticulous pre-procedural planning and customized device strategies. Careful patient selection based on MR etiology, proportionality, ventricular function, and anatomical suitability is essential for optimizing outcomes in this rapidly evolving field.

## 1. Introduction

Mitral regurgitation (MR) remains one of the most prevalent and challenging valvular heart diseases globally, especially in aging populations with increasing comorbidities. MR has traditionally been managed with medical therapy and surgical repair or replacement. However, the advent of transcatheter approaches has significantly expanded treatment options. These innovations offer life-saving interventions to patients previously deemed inoperable due to high surgical risk. In recent years, mitral transcatheter edge-to-edge repair (MTEER) and transcatheter mitral valve replacement (TMVR) technologies have rapidly evolved. These advances have expanded treatment options for both primary and secondary MR. This review provides a comprehensive update on current transcatheter therapies for MR, emphasizing their mechanisms, evidence base, and evolving clinical roles.

## 2. Mitral Regurgitation: A Powerful Prognostic Indicator in Heart Failure

The development of MR can be the result of the disruption of any part of the valvular apparatus (leaflets, annulus, chordae, papillary muscles). It can be either primary (PMR), due to intrinsic valve disease, or secondary (SMR), resulting predominantly from left ventricular dysfunction and dilation, but also from left atrial dilatation. The Carpentier classification provides a systematic approach to categorizing MR based on the underlying mechanism of valve dysfunction. It classifies MR into three types: Type I, characterized by normal leaflet motion with annular dilation or leaflet perforation; Type II, defined by excessive leaflet motion due to chordal elongation or rupture, as seen in mitral valve prolapse; and Type III, which involves restricted leaflet motion, either during diastole (Type IIIa, due to rheumatic disease) or systole (Type IIIb, often secondary to left ventricular dysfunction in heart failure). This classification is essential for guiding therapeutic decisions, as it helps differentiate primary from secondary MR and determines the most appropriate surgical or interventional approach.

MR plays a significant role in the progression and severity of heart failure (HF), contributing to increased morbidity and mortality [1]. As a common valvular abnormality, MR increases left atrial pressure and promotes ventricular remodeling. These changes exacerbate HF symptoms such as dyspnea and fatigue. For SMR in particular, the prognostic implications are crucial. In a meta-analysis comprising 53 studies and 45,900 patients, the presence of SMR was associated with increased all-cause mortality (risk ratio (RR) 1.79, 95% confidence interval (CI) 1.47–2.18), hospitalization for HF (HHF) (RR 2.26, 95% CI 1.92–2.67), and cardiac mortality (RR 2.62, 95% CI 1.87–3.69) [2].

Treatment options for MR include guideline-directed medical therapy (GDMT), cardiac resynchronization therapy, and structural interventions. Clinical trials have shown a reduction in SMR with HF medication, namely angiotensin receptor neprilysin inhibitors (ARNIs) and sodium-glucose co-transporter-2 inhibitors (SGLT2i) [3, 4]. However, a sub-analysis of a recent randomized trial in patients with HF and SMR showed that many patients are unable to tolerate one or more drug classes or achieve target doses [5]. Standard cardiac resynchronization therapy and conduction system pacing are also a mainstay in the management of SMR when dyssynchrony is present, as confirmed by a recent meta-analysis showing a reduced MR prevalence post-device implantation [6].

In an attempt to further decrease the excess morbidity and mortality associated with MR, research efforts have focused on the invasive correction of MR through transcatheter approaches. Table 1 summarizes representative transcatheter devices currently used or under investigation. However, the list is not exhaustive, as numerous TMVR platforms are currently in different stages of clinical development worldwide.

**Table 1.** **Transcatheter approaches for the management of MR**.

Device (Manufacturer)	Mechanism	MR type	Access	Technical complexity	Procedural duration	Main advantages	Limitations-complications
MitraClip (Abbott)	MTEER	Secondary MR	TF-TS	Moderate	60–120 min	Extensive clinical data	Severe leaflet calcification
		Degenerative MR with focal prolapse				Minimally invasive	Short posterior leaflet
						Widely available	Large flail gap (>10 mm)
							Commissural jets
PASCAL	MTEER	Complex degenerative MR	TF-TS	Moderate-High	90–150 min	More leaflet-friendly than MitraClip	Severe annular calcification in grasping zone
(Edwards Lifesciences)		Functional MR with wide gap				Better suited for large coaptation gaps	Very small mitral valve area
DragonFly (Valgen MedTech)	MTEER	Degenerative MR	TF-TS	Moderate–high	90–110 min	Central compressible filler with independent leaflet capture	Severe leaflet calcification in grasping zone
							Leaflet length <7 mm
							MVA <3.5 cm^2^
ValveClamp (Hanyu Medical)	MTEER	Degenerative and functional MR	TA	High	60–120 min	Direct transapical access providing improved coaxial alignment with the mitral valve and facilitating stable leaflet capture	Severe leaflet calcification in grasping zone
							Insufficient leaflet length for grasping
							Small mitral valve area with risk of mitral stenosis
Carillon (Cardiac Dimensions)	Indirect annuloplasty via coronary sinus	Secondary MR	TJ	Low-Moderate	45–90 min	Preserves native valve	Severe MR
						Minimally invasive	Coronary sinus-circumflex artery proximity issues
						Suitable for SMR	
							Primary MR
Cardioband (Edwards Lifesciences)	Direct annuloplasty, cinching mitral annulus	Secondary MR with annular dilation	TF-TS	High	120–180 min	Mimics surgical annuloplasty	Extensive annular calcification
						Adjustable post-implantation	Posterior annular anatomy unsuitable for anchoring
Tendyne (Abbott)	TMVR	Severe degenerative or mixed MR	TA	High	120–180 min	Complete MR elimination	Small LV cavity (LVOT obstruction risk)
						Fully repositionable and retrievable	
		MAC-associated MR					High surgical risk for apical access
Intrepid (Medtronic)	TMVR	Functional MR with large annulus	TA	High	120–180 min	No need for annular anchoring	LVOT obstruction risk
			TF-TS			Suitable for diverse anatomies	Severe small LV cavity
Sapien M3 (Edwards Lifesciences)	TMVR	Degenerative MR	TF-TS	High	120–150 min	Fully percutaneous transseptal delivery	Requires precise docking
							Small LVOT
						High procedural success	Thrombosis risk
						Durable MR elimination	
						Allows future valve-in-valve reintervention	

MTEER, mitral transcatheter edge-to-edge repair; TMVR, transcatheter mitral valve replacement; TF-TS, transfemoral–transseptal; TJ, transjugular; TA, transapical; MR, mitral regurgitation; SMR, secondary mitral regurgitation; MVA, mitral valve area; MAC, mitral annular calcification; LV, left ventricular; LVOT, left ventricular outflow tract.

## 3. Transcatheter Interventions

### 3.1 Edge-to-Edge Repair

MTEER is perhaps the most decorated method of percutaneous mitral valve intervention. The technique involves the percutaneous placement of a device, such as the MitraClip (Abbott, IL, USA) or the PASCAL (Edwards Lifesciences, CA, USA), to approximate the mitral valve leaflets, reducing regurgitant flow. By mimicking the surgical Alfieri stitch, MTEER creates a double-orifice valve, enhancing coaptation and restoring more efficient valve function.

#### 3.1.1 MITRA-FR and COAPT Trials: 2 Sides of the Same Coin

The MITRA-FR and COAPT trials were two landmark randomized controlled studies that evaluated the role of MTEER using the MitraClip in patients with SMR and HF. While both trials sought to determine the efficacy of MitraClip compared to GDMT alone, there were important differences in patient selection and trial design (Table 2). These differences meant that MITRA-FR included patients with proportionate MR (where MR severity matched the degree of LV dysfunction), whereas COAPT focused on patients with disproportionate MR, meaning MR was excessively severe relative to the degree of LV dysfunction. The results of the two trials diverged significantly. MITRA-FR found no significant benefit of MitraClip over GDMT [7], while COAPT showed a significant benefit with MitraClip compared to GDMT alone [8]. These findings highlight the importance of careful patient selection when considering MTEER for SMR. The concept of proportionate vs. disproportionate MR has since become a crucial framework for deciding which patients will derive the greatest benefit from MitraClip therapy [9]. However, even though it provides a useful conceptual explanation for the divergent results of MITRA-FR and COAPT, patient selection for MTEER should not rely on this concept alone. Instead, treatment decisions should integrate MR severity, left and right ventricular size and function, and anatomical suitability, among others, within a multidisciplinary Heart Team evaluation.

**Table 2.** **Key characteristics of the MITRA-FR and the COAPT trials**.

	MITRA-FR	COAPT
Sample size	304 patients	614 patients
LVEF inclusion criteria	≤50%	20–50%
EROA	≥20 mm^2^	≥30 mm^2^
LVEDV	Larger (135 ± 35 mL/m^2^)	Smaller (101 ± 34 mL/m^2^)
Severity of MR	Moderate-to-severe	Severe
GDMT	Less rigorously optimized	Strictly optimized and monitored
Primary endpoint	All-cause death or unplanned HF hospitalization	HF hospitalization at 24 months
Outcome	No difference in mortality or HF hospitalizations	Significant reduction in both
Interpretation	MR proportional to LV dysfunction (less likely to benefit from MTEER)	Disproportionate MR (more likely to benefit)

LVEF, left ventricular ejection fraction; EROA, effective regurgitant orifice area; LVEDV, left ventricular end-diastolic volume; GDMT, guideline-directed medical therapy; HF, heart failure; MR, mitral regurgitation; MTEER, mitral transcatheter edge-to-edge repair.

Recently, the RESHAPE-HF2 trial assessed the efficacy of MTEER using MitraClip in symptomatic HF patients with grade 3+/4+ SMR despite maximally tolerated GDMT (Table 3) [10]. A total of 505 patients (mean age 70 years, 20% female, median LVEF 32%) were randomized to MTEER + GDMT (n = 250) or GDMT alone (n = 255) with a median follow-up of 18.8 months [10]. The primary outcome, a composite of first or recurrent HF hospitalization or cardiovascular death at 24 months, was significantly lower in the MTEER group (37.0 vs. 58.9 events per 100 patient-years, RR 0.64, number needed to treat = 5.1) [10]. Recurrent HF hospitalizations, combined cardiovascular hospitalizations and all-cause mortality, and days lost to death or HF hospitalization were also reduced with MTEER [10]. At 12 months, MR ≤2+ was achieved in 90.4% of MTEER patients versus 36.1% in the GDMT group. Patient-centered, quality of life outcomes (change in functional class, change in Kansas City Cardiomyopathy Questionnaire (KCCQ)) also favored MTEER [10]. All-cause and cardiovascular mortality were numerically lower in the MTEER arm, though not statistically significant [10]. Patients with prior HF hospitalizations derived the most benefit from MTEER, particularly in reducing recurrent events [11]. Notably, despite a baseline EROA (0.23 cm^2^) more aligned with MITRA-FR than COAPT, MTEER demonstrated superiority over GDMT, reinforcing its role in appropriately selected SMR patients.

**Table 3.** **Key clinical trials evaluating transcatheter mitral interventions and their principal outcomes**.

Trial	Device	Design	Population	N	Primary outcome	Key result
MITRA-FR	MitraClip vs SoC	RCT	Secondary MR + HF	304	Death or HF hospitalization	OR 1.16 (no benefit vs GDMT)
COAPT	MitraClip vs SoC	RCT	Secondary MR + HF	614	HF hospitalization	HR 0.53
CLASP IID	PASCAL vs MitraClip	RCT	Degenerative MR	300	MR ≤2+ at 1 year	95.8% vs 93.8%
DRAGONFLY-DMR	DragonFly	Prospective, single-arm	Degenerative MR	112	Freedom from all-cause mortality, mitral valve reintervention, and MR >2+ at 1 year	87.5%
REDUCE-FMR	Carillon	RCT	Secondary MR	120	LVESV reduction	–7.1 mL vs control
ENCIRCLE	Sapien M3	Prospective, single-arm trial	Severe MR unsuitable for MTEER	299	Death or HF hospitalization	25.2% at 1 year

RCT, randomized controlled trial; SoC, standard of care; GDMT, guideline-directed medical therapy; HF, heart failure; MR, mitral regurgitation; LVESV, left ventricular end-systolic volume; OR, odds ratio; HR, hazard ratio.

#### 3.1.2 The PASCAL System: An Alternative MTEER Approach

The PASCAL transcatheter valve repair system is an alternative to the MitraClip for MTEER in patients with significant MR. Developed as a next-generation device, PASCAL offers key design modifications aimed at improving procedural outcomes, particularly in patients with complex mitral anatomy or challenging leaflet morphology. The PASCAL device is wider (10 mm vs. MitraClip’s 6 mm) and features a central spacer, which fills the regurgitant orifice. This helps distribute forces across the valve and reduces leaflet stress. At the same time, the MitraClip does not have a spacer, relying entirely on leaflet coaptation, which may be less effective in certain anatomies. Moreover, PASCAL has elongated, atraumatic paddles and clasps designed to minimize leaflet tension and improve leaflet capture, while MitraClip’s shorter, rigid arms may exert more stress on the leaflets, particularly in fragile or highly mobile valves.

The CLASP IID (Edwards PASCAL Transcatheter Valve Repair System Pivotal Clinical Trial) was the first study directly comparing PASCAL versus MitraClip in patients with degenerative mitral regurgitation (DMR) who were at high surgical risk [12]. It was a multicenter, prospective, randomized controlled trial conducted in the United States and Europe. It enrolled 300 patients with severe symptomatic DMR. PASCAL (95.8%) and MitraClip (93.8%) achieved MR ≤2+ at 1 year, demonstrating comparable efficacy. Slightly higher rates of MR ≤1 were achieved with PASCAL (77.1% vs. 71.3% with MitraClip). Similar rates of major adverse events were noted in both groups.

Importantly, the PASCAL MTEER system could be safely and effectively deployed in patients once deemed unsuitable for MTEER based on anatomic characteristics, namely, presence of ≥2 independent significant jets, evidence of severe bileaflet/multi scallop prolapse involvement, mitral valve orifice area <4.0 cm^2^, large flail gap/large flail width, presence of 1 significant jet in the commissural area, presence of significant cleft or perforation in the grasping area, moderate to severe calcification in the grasping area, leaflet mobility length <8 mm, history of endocarditis and significant tissue defects in the leaflet, and flail width >15 mm and/or flail gap >10 mm [13]. Although this was the first randomized comparison between the PASCAL and MitraClip systems in degenerative mitral regurgitation, enrolled patients were deemed at prohibitive surgical risk, thereby limiting generalizability to lower-risk populations. Furthermore, while a 2-year follow-up demonstrates sustained MR reduction and favorable clinical outcomes [14], longer-term durability beyond this period remains uncertain, and continued follow-up is ongoing.

In its post-marketing MiCLASP study [15], the PASCAL system reproduced its efficacy and safety in patients with significant MR of any etiology. Specifically, incidence rates of major adverse events at 30 days were acceptable (6.8%), with sustained MR reduction at 1 year (≤2: 98%, ≤1: 82.6%), 87.3% 1-year survival, and 84.3% 1-year freedom from HF hospitalization. These findings were paired with improvements in quality-of-life and functional capacity metrics.

Continuous improvement of PASCAL technology is also critical to the success of the device. The REPAIR study was a multicenter, observational registry that included 2165 patients treated with the PASCAL system across 14 centers in Germany from 2019 to 2024 [16]. Unlike randomized controlled trials, REPAIR did not apply strict inclusion/exclusion criteria, making it a real-world evaluation of PASCAL’s performance. Patients were categorized into three groups based on the iteration of the PASCAL system used: P10-only cohort (first-generation PASCAL device), P10/AceGen1 cohort (introduction of the smaller PASCAL Ace device), P10/AcePrec cohort (introduction of the PASCAL Precision delivery system). The primary endpoint was the achievement of MR ≤1+ at discharge. Secondary endpoints included technical success rate of the procedure, durability of MR reduction at 30 days, 1 year, and 2 years, reintervention rates, and functional outcomes (NYHA class improvement, quality of life). The technical success rate was 97.0% overall, with no significant differences between the three PASCAL iterations. The PASCAL Ace device (smaller size) and Precision delivery system (improved control and positioning) have led to higher MR ≤1+ rates at discharge. The 77% MR ≤1+ rate with PASCAL Precision is the highest reported for any PASCAL iteration, making it the most optimized iteration so far. In a prospective study involving 66 consecutive patients with grade 3+/4+ MR (predominantly functional etiology), the device demonstrated a 98.5% procedural success rate [17]. At a median follow-up of 5 months, 84.7% of patients had MR grade ≤1, accompanied by significant reverse cardiac remodeling (LVEF improved from 45% to 53%) [17]. MTEER with PASCAL Ace also resulted in meaningful functional and symptomatic improvement assessed by NYHA class, 6-minute walk distance, KCCQ score, NT-proBNP levels, and pulmonary capillary wedge pressure [17]. At 5 months, mortality was low (3 deaths, none cardiovascular) [17], supporting the safety and efficacy of PASCAL Ace in a real-world cohort.

#### 3.1.3 Other MTEER Devices

Another transcatheter edge-to-edge repair system that has recently emerged is the DragonFly (Valgen MedTech). The system is delivered via a transfemoral transseptal approach and consists of a steerable delivery system and a clip implant available in multiple sizes to accommodate varying mitral anatomies [18]. A distinctive feature of the device is the presence of a central compressible filler, which expands within the regurgitant orifice when the device arms are closed, enhancing leaflet coaptation while potentially limiting excessive leaflet tension [18]. In addition, the device allows independent leaflet grasping and controlled arm angulation, which may facilitate treatment of complex mitral anatomies and improve device positioning [18]. Early clinical experience has demonstrated encouraging procedural and short-term outcomes. In a multicenter first-in-human study involving patients with severe MR at high surgical risk, device implantation was successful in all treated patients, with MR ≤2+ achieved in 100% of cases at discharge and sustained symptomatic improvement at 30 days [18]. Subsequent evaluation in the DRAGONFLY-DMR multicenter trial further supported the safety and effectiveness of this platform in patients with degenerative MR deemed unsuitable for surgery [19]. At 1 year, the trial reported a clinical success rate of 87.5%, with MR ≤2+ maintained in approximately 92% of patients, alongside significant improvements in functional status and quality-of-life measures [19].

Another MTEER platform developed in China is the ValveClamp system (Hanyu Medical Technology), which is performed through a transapical approach. The system consists of a clamp device delivered through a 16-Fr introducer sheath and incorporates a spherical valve-crossing device designed to prevent entanglement within the chordae tendineae during advancement across the mitral valve [20]. The clamp features V-shaped matching arms and a closed-ring mechanism that facilitates leaflet capture and stabilization while creating a double-orifice mitral valve configuration [20]. The transapical route provides a short and direct trajectory to the mitral valve, potentially improving coaxial alignment and procedural control compared with transseptal approaches [20]. Early feasibility studies have demonstrated encouraging procedural performance. In a prospective series of patients with functional MR, device implantation was successful in 100% of cases, with most patients requiring a single clamp and a mean procedural time of approximately 60 minutes. MR reduction to ≤2+ was achieved in all patients at follow-up, accompanied by improvements in ventricular remodeling and functional status [20]. Subsequent multicenter data further supported the safety and feasibility of this approach. In a prospective registry of patients undergoing transapical ValveClamp implantation for secondary MR, technical success was achieved in all patients, with sustained MR reduction and significant improvement in NYHA functional class and quality-of-life measures at one year [21]. Longer-term observational data have also demonstrated favorable outcomes, with durable MR reduction and improvements in ventricular dimensions and pulmonary pressures at 2-year follow-up [22].

Despite encouraging early results, the available evidence for those emerging MTEER platforms remains limited. Data arises from non-randomized studies, which restricts the ability to determine comparative efficacy relative to established devices such as MitraClip or PASCAL. In addition, enrolled patients were typically highly selected, often consisting of individuals with degenerative MR at high or prohibitive surgical risk and with favorable mitral valve anatomy suitable for leaflet-based repair. Such selection criteria may introduce referral and anatomical selection bias and limit the generalizability of these findings to broader MR populations encountered in routine practice. Furthermore, follow-up durations remain relatively short, and longer-term data are needed to assess device durability, recurrent MR, and the need for reintervention.

### 3.2 Annuloplasty-Based Interventions

#### 3.2.1 Carillon Mitral Contour System

The Carillon Mitral Contour System (Cardiac Dimensions, WA, USA) is a percutaneous indirect annuloplasty device designed to treat SMR by reshaping the mitral annulus. Unlike leaflet-based MTEER systems, the Carillon device focuses on annular reduction, leveraging the anatomical relationship between the coronary sinus and the mitral annulus. By implanting a self-expanding nitinol device within the coronary sinus, Carillon applies external compression to reduce annular dilation and improve leaflet coaptation. This minimally invasive approach is particularly appealing for patients with HF and FMR, as it preserves native valve anatomy and allows for future interventions if needed.

The feasibility of this technology was proven more than a decade ago, through the AMADEUS study of 30 patients receiving this device, demonstrating acceptable major adverse event rates and improvement in quality of life [23]. The REDUCE-FMR was the first sham-controlled trial evaluating its efficacy in improving SMR, involving symptomatic patients with impaired LVEF, LV end-diastolic diameter >55 mm, at least moderate SMR, on stable GDMT [24]. The device was implanted successfully in 84% of the study group. Compared to the control group, patients treated with the Carillon system exhibited a significant reduction in MR severity, coupled with lower LV volumes [24]. Even in patients with severe LV enlargement (LV end-diastolic diameter >65 mm), this therapeutic option remains valid, with similar outcomes to less dilated LVs [25]. An individual patient data meta-analysis, including REDUCE-FMR, TITAN, and TITAN II confirmed the benefits of this intervention through MR reduction, left atrial volume reduction, LV reverse remodeling, ultimately translating into quality of life improvements and lower rates of HF hospitalization [26]. Finally, the CINCH registry evaluated the Carillon device in real-world SMR patients, confirming MR reduction, symptom improvement, reduction of major adverse cardiovascular event rates, and safety over five years, with potential benefits for broader patient populations, including HF with preserved ejection fraction (HFpEF) [27].

#### 3.2.2 Cardioband

The Cardioband Mitral System (Edwards Lifesciences, CA, USA) is a transcatheter direct annuloplasty device designed to treat functional SMR by reducing mitral annular dilation and restoring leaflet coaptation. Unlike indirect annuloplasty systems such as Carillon, Cardioband is implanted directly onto the mitral annulus via a transfemoral, transseptal approach. The device consists of an adjustable, implantable band that is anchored along the posterior annulus using multiple fixation screws. Once securely placed, the band is incrementally cinched, effectively reducing annular size and improving mitral valve function. As with the Carillon system, this technique mimics surgical annuloplasty, preserving the native valve and allowing for future interventions if needed. Cardioband is particularly suited for patients with annular dilation and preserved leaflet mobility, offering a physiological and customizable approach to MR reduction while minimizing procedural risks compared to surgery.

The Cardioband feasibility study evaluated the safety and efficacy of the Cardioband transcatheter mitral annuloplasty system in high-risk patients with SMR [28]. Conducted across five European centers, the study enrolled 31 patients with moderate-to-severe or severe MR despite optimal medical therapy. The Cardioband was successfully implanted in all patients, with a 93.6% technical success rate. Adjustment of the device led to a significant reduction in septolateral annular dimension (from 36.8 mm to 29.0 mm, *p* < 0.01), translating into effective MR reduction. At 30 days, 88% of patients had MR ≤2+, and no procedural mortality occurred. The safety profile was favorable, with an in-hospital mortality rate of 6.5%, though unrelated to the device. These early results demonstrated that percutaneous direct annuloplasty with Cardioband is feasible and safe.

A subsequent study evaluated the 1-year outcomes of transcatheter mitral valve repair using the Cardioband system in patients with moderate-to-severe or severe SMR [29]. Conducted across 11 European centers, the study enrolled 60 patients who were symptomatic despite receiving GDMT. The technical success rate was 97%, and device success, based on Mitral Valve Academic Research Consortium (MVARC) criteria, was 72%. Over one year, MR reduction was achieved in most patients (95% had MR ≤ moderate at 12 months), though 22% experienced MR worsening by at least one grade. Clinical outcomes showed significant improvements in NYHA class (79% in class I/II at 1 year vs. 14% at baseline), quality of life (–19 points in Minnesota Living with Heart Failure Questionnaire), and exercise capacity (+58 meters in 6-minute walk test). Survival at one year was 87%, with 66% free from HF hospitalization and 78% free from reintervention. Device refinements during the study addressed anchor disengagement issues, improving procedural durability. Last but not least, despite the lack of randomized trials comparing this annuloplasty technique with the MTEER, a propensity score-matched study found a greater reduction in clinical endpoints (HF hospitalization, mortality), as well as greater improvements in quality of life at the 12-month follow-up in favor of the Cardioband system [30]. However, these findings should be interpreted cautiously. The analysis was based on a propensity score-matched observational cohort rather than a randomized comparison, and residual confounding and selection bias may persist despite statistical adjustment. In addition, the multicenter registry design without core-laboratory adjudication and the inclusion of predominantly high-risk patients may limit the generalizability of these results to broader MR populations. Overall, the Cardioband system demonstrates reasonable performance and safety, with randomized trials needed to further assess its benefits.

### 3.3 Transcatheter Mitral Valve Replacement

Transcatheter mitral valve replacement (TMVR) is an emerging therapy for patients with significant MR who are poor candidates for conventional surgery, or in cases of MTEER failure/unsuitability. For MTEER specifically, anatomical constraints should be acknowledged, such as severe mitral annular dilation, short posterior leaflet, extensive calcification, or severe leaflet restriction where leaflet coaptation cannot be restored adequately. It may also be considered in cases of recurrent or residual MR after MTEER, where another MTEER procedure is unlikely to succeed. Current TMVR devices, such as Tendyne (Abbott, IL, USA), Intrepid (Medtronic, MN, USA), Sapien M3 (Edwards Lifesciences, CA, USA), and Evoque (Edwards Lifesciences, CA, USA), are expanding treatment options for these complex cases. However, dedicated TMVR systems remain investigational, and currently available evidence largely reflects early feasibility studies and registries, emphasizing procedural success and short-term outcomes rather than long-term efficacy and durability.

#### 3.3.1 Pre-Procedural Planning

Transesophageal echocardiography (TEE), particularly three-dimensional (3D) TEE, plays a pivotal role in patient selection and procedural planning for TMVR by providing high-resolution visualization of the mitral valve apparatus and surrounding structures [31]. A comprehensive pre-procedural TEE assessment includes evaluation of mitral annular dimensions, leaflet morphology and mobility, commissural anatomy, and subvalvular structures, which may influence device feasibility and procedural complexity [31]. 3D-TEE allows dynamic assessment of the mitral annulus throughout the cardiac cycle, with measurements typically obtained at end-systole when annular dimensions are maximal [31]. In addition, TEE contributes to risk stratification for left ventricular outflow tract (LVOT) obstruction by assessing baseline LVOT area, anterior mitral leaflet length, septal thickness, and the mitral–aortic angle [31]. Functional parameters, including left and right ventricular function, pulmonary pressures, and left atrial size, are also important components of pre-procedural evaluation, as they influence procedural feasibility and clinical outcomes [31]. Furthermore, TEE enables detailed assessment of the interatrial septum and optimal transseptal puncture site, which is critical for achieving appropriate trajectory and device coaxiality during transseptal TMVR procedures [31].

Cardiac multidetector computed tomography (CT) plays a central role in pre-procedural planning for TMVR by providing comprehensive anatomical and procedural guidance. It enables precise characterization of mitral annular geometry (annular area/perimeter, intercommissural and anteroposterior measurements) to assist device sizing and appropriate oversizing to prevent paravalvular leak or device embolization [32]. The mitral annulus is typically modeled using a 3D saddle-shaped or D-shaped reconstruction to account for its complex geometry. In addition, CT allows detailed assessment of the mitral leaflets and subvalvular apparatus, including anterior mitral leaflet length and papillary muscle insertion, which may influence device anchoring and contribute to left ventricular outflow tract (LVOT) obstruction. A major component of CT-based planning is the simulation of virtual valve implantation to estimate the predicted neo-LVOT area, a key determinant of post-procedural LVOT obstruction risk, with values below approximately 1.7–2.0 cm^2^ associated with higher risk. Other anatomical predictors assessed by CT include septal thickness, left ventricular geometry, and the aorto-mitral angle. Furthermore, CT is essential for evaluating mitral annular calcification (MAC) distribution, thickness, and circumferential extent to determine the adequacy of the landing zone and anchoring stability. Finally, CT provides procedural guidance by enabling planning of the optimal transapical or transseptal access trajectory and determining fluoroscopic projection angles to facilitate coaxial device deployment.

#### 3.3.2 Tendyne

Tendyne represents a promising transcatheter solution for patients unsuitable for repair, expanding treatment options for high-risk MR patients who previously had limited surgical alternatives. It is a self-expanding, bioprosthetic valve made of a tri-leaflet porcine pericardial valve within a nitinol frame [33]. It is delivered via a transapical approach through a small left thoracotomy [33]. The system is fully repositionable and retrievable before final deployment, allowing for optimal placement. Unlike surgical mitral valve replacement, Tendyne is secured using an apical tether, which stabilizes the valve within the native annulus and prevents migration [33]. This design accommodates a wide range of mitral anatomies, including patients with functional, degenerative, or mixed MR, as well as those with severe annular dilation or MAC.

Several disadvantages should be acknowledged. The implantation requires a mini-thoracotomy and transapical access, which increases procedural risk compared to transfemoral techniques. It should be stressed that, due to the apical puncture, there is an increased risk of bleeding, pericardial effusion, and tamponade. The apical tethering system exerts tension on the LV myocardium, potentially contributing to LV remodeling or dysfunction over time. Furthermore, Tendyne, like other TMVR devices, can obstruct the LVOT, leading to hemodynamic instability or severe obstruction, with patients having a small LV cavity size or a prominent anterior mitral leaflet being at higher risk.

The Tendyne Global Feasibility Trial evaluated the safety and efficacy of the Tendyne system in high-risk patients with symptomatic MR [34]. The technical success rate was 93.3%, with successful valve implantation in 28 out of 30 patients. At 30 days, 96.2% of patients had complete MR elimination, with only one patient experiencing mild residual MR. Additionally, left ventricular end-diastolic volume significantly decreased, suggesting positive left ventricular remodeling. The safety profile was favorable, with no intraprocedural deaths, strokes, or myocardial infarctions. The 30-day freedom from major adverse events was 83.3%, and only 13.8% required rehospitalization for HF. The Tendyne 2-Year Outcomes Study showed sustained MR elimination in 93.2% of surviving patients, with no cases of greater than mild MR [35]. The technical success rate was 97%, and HF hospitalization rates were significantly reduced. All-cause mortality was 39% at 2 years, with most deaths (43.6%) occurring within the first 90 days, reflecting the high-risk nature of the population. The safety profile remained acceptable, with low rates of stroke (5%), endocarditis (5%), and structural valve deterioration (0%).

Along the same lines, the TENDER study, a real-world, European multicenter registry evaluating transapical mitral valve implantation with the Tendyne system in high-risk patients (median age 77 years, 40% female) with symptomatic moderate-to-severe MR of any etiology showcased a technical success rate of 95%, with MR reduced to ≤1+ in 98% of successfully implanted patients [36]. Pulmonary artery pressure and tricuspid regurgitation severity also significantly decreased post-procedure [37]. At 1 year, 83% of patients were in NYHA class I/II, while all-cause and cardiovascular mortality rates were 29% and 17%, respectively [36]. Reintervention or surgery was required in 4%. Importantly, HF hospitalizations significantly decreased compared to the prior year [36].

#### 3.3.3 Intrepid

The Intrepid system is a TMVR device composed of a bioprosthesis and a transapical delivery system [38]. The bioprosthesis features a trileaflet bovine pericardial valve housed in a dual-structure nitinol frame, with an inner circular stent for valve support and a flexible outer fixation ring designed to conform to the dynamic anatomy of the mitral annulus [38]. This outer ring provides fixation and sealing via its variable radial stiffness and a champagne cork-like shape that resists migration, while a brim aids imaging and tissue ingrowth [38]. The valve, available in various outer diameters, is designed to preserve native structures and minimize outflow obstruction [38]. Covered in polyester fabric, it prevents leaks and promotes long-term integration [38]. The transapical delivery system uses hydraulic actuation for precise, rotation-free deployment, eliminating the need for leaflet capture [38].

The study by Bapat *et al*. [39] reported the early clinical experience with the Intrepid TMVR system in patients who were deemed high or extreme risk for cardiac surgery. Conducted across 14 international centers, the pilot study enrolled 50 patients (mean age 73), with 86% in NYHA class III or IV, and most having SMR. The TMVR device was successfully implanted in 48 patients (96%). At 30 days, mortality was 14%, primarily due to bleeding or HF, but all surviving patients showed significant symptomatic improvement and mild or no residual MR [39]. The device showed no malfunction, thrombosis, or structural valve degeneration during a median follow-up of 173 days [39]. A subsequent cohort study followed up 252 patients treated with transapical Intrepid implantation, reporting all-cause mortality rates of 13.1%, 27.3%, and 36.2% at 30 days, 1 year, and 2 years, respectively [40]. Bleeding events were documented in 22.3% of the participants at 30 days [40]. Importantly, at 2 years, most of the alive participants were in good functional status (82.1% in NYHA class I/II) and had at most mild MR [40]. Over five years, the early Intrepid transapical TMVR device provided durable valve performance, sustained elimination of MR, and lasting symptom improvement in those high-risk patients, despite high overall mortality reflecting the population’s advanced disease [41].

Lately, a newer-generation, 35-F transfemoral transseptal access system has been introduced in the delivery of the Intrepid TMVR system. The study by Zahr *et al*. [42] presented 1-year results from the Early Feasibility Study of this approach in patients with symptomatic moderate-to-severe or severe MR at high surgical risk. Conducted at nine U.S. centers, 33 patients were enrolled, with 94% successfully receiving valve implants. At 30 days, there were no deaths or strokes, though 24% experienced vascular complications [42]. At 1 year, all-cause mortality was 6.7%, MR was nearly eliminated in all patients, and 92% of survivors were in NYHA class I or II, reflecting sustained symptom improvement [42]. Quality of life scores improved significantly, and valve performance remained stable, with low gradients and minimal regurgitation [42]. While vascular complications and venous thromboembolism were noted, no strokes or device-related deaths occurred [42]. These findings suggest that the transfemoral transseptal Intrepid TMVR system offers a promising, less invasive alternative for high-risk patients, with durable hemodynamic and clinical benefits.

#### 3.3.4 Sapien M3

The SAPIEN M3 system (Edwards Lifesciences) is a fully percutaneous, transseptal TMVR platform designed to treat severe, symptomatic MR in patients considered unsuitable for surgery or MTEER. The system uses a novel two-component design: a nitinol “dock” that encircles and stabilizes the subvalvular apparatus to create a secure landing zone, followed by implantation of a balloon-expandable SAPIEN M3 valve within this dock. This approach aims to overcome anatomical challenges that limit MTEER success, avoid the morbidity of transapical access, and provide a repeatable, repositionable platform that allows for future valve-in-valve reintervention if needed.

In the prospective, multicenter ENCIRCLE pivotal trial, the first large-scale evaluation of a fully percutaneous transseptal TMVR system, 299 patients underwent attempted implantation, with device success in 96% of cases [43]. These were elderly, high-risk patients (median age 77 years, mean STS-PROM 6.6%) with predominantly severe (grade 4) MR and significant comorbidity burden [43]. Clinical outcomes at one year demonstrated safety and effectiveness: the primary composite endpoint of all-cause mortality or HF rehospitalization was 25.2%, significantly outperforming the prespecified 45% benchmark based on contemporary medical therapy trials [43]. Mortality at one year was 13.9%, 30-day mortality was only 0.7%, and there were no intraprocedural deaths or conversions to surgery [43]. MR reduction was durable, with 96% of patients achieving mild or less MR at one year, accompanied by meaningful improvements in NYHA class (88% in class I-II at one year) and quality of life (mean +18-point KCCQ improvement) [43]. Rates of disabling stroke (3.9%), valve thrombosis (6.7%), and reintervention (6.4%) were notable but manageable within this high-risk cohort [43]. However, as a prospective single-arm study in patients unsuitable for surgery or MTEER without a randomized comparator group, the ability to draw definitive conclusions regarding comparative efficacy and safety is restricted. Additionally, the trial population was highly selected based on strict anatomical and clinical eligibility criteria, potentially limiting applicability to broader real-world cohorts of patients with MR. Larger studies with longer follow-up are required to better establish its long-term safety and efficacy.

#### 3.3.5 Valves in Development

Several additional TMVR systems are currently under clinical investigation, highlighting the rapidly evolving landscape. The AltaValve system (4C Medical Technologies, MN, USA) is a transapical or transseptal supra-annular TMVR device, designed to reduce the risk of LVOT obstruction by positioning the prosthesis above the mitral annulus, minimizing interaction with subvalvular structures [44]. In an early feasibility study and compassionate use series, 14 patients (mean age 77.9 years, 71% women, mean STS score 5.4%) with severe symptomatic MR, including those with atrial functional MR, were treated with AltaValve [45]. Eleven underwent transseptal and three underwent transapical implantation. The procedure achieved technical success and MR reduction to none/trace in all cases and there were no instances of LVOT obstruction [45]. At 30 days, all-cause mortality was 14%, and NYHA class III/IV symptoms were eliminated (reduced from 79% to 0%) [45]. Among surviving patients, 91% had no or trace MR and 9% had mild MR [45], supporting the feasibility of this supra-annular TMVR approach. Another feasibility study showed that the AltaValve TMVR system can be implanted safely and effectively, providing high procedural success, sustained MR reduction, and improved symptoms and quality of life over six months in high-risk patients unsuitable for surgery [46]. Clinical experience with AltaValve remains limited, however, and future studies are needed to assess long-term durability, thrombotic risk, and clinical outcomes beyond short-term follow-up.

The Cephea TMVR system (Abbott, IL, USA) is a fully percutaneous, transseptally delivered valve designed to accommodate a wide range of mitral anatomies using an atrial anchoring mechanism and a self-expanding nitinol frame. Its unique design avoids entanglement with subvalvular structures, aiming to minimize LVOT obstruction and paravalvular leak. A preclinical study has demonstrated promising feasibility and safety. In a chronic bovine model, surgical antegrade transatrial implantation showed excellent hemodynamic performance over 30–90 days, with stable valve position, minimal paravalvular leak, no LVOT obstruction, and favorable healing with no thrombosis or endocarditis [47]. Additionally, a dedicated transseptal delivery system was acutely tested in a porcine model, confirming accurate positioning and excellent immediate valve performance on echocardiography and autopsy [47]. These experimental results were followed by a first-in-human series involving three elderly patients (mean age 79 years) with severe PMR who were at prohibitive surgical risk and unsuitable for MTEER due to anatomic factors [48]. Successful transfemoral-transseptal valve implantation was achieved in all cases, with no procedural complications [48]. Post-implant, valve function remained normal with a mean gradient of 3 mmHg, no significant regurgitation, and no LVOT obstruction [48]. At 6-month follow-up, quality of life improved significantly, and imaging confirmed valve durability, geometric stability, and absence of structural deterioration [48]. An ongoing feasibility study (NCT05061004) along with additional evidence is, however, required.

The HighLife system (Highlife SAS, Paris, France) employs a dual-component design, combining a subannular ring delivered via the transfemoral arterial route with a valve prosthesis implanted transapically [49]. This configuration aims to enhance anchoring stability and reduce the risk of valve embolization. In a prospective, multicenter, nonrandomized feasibility study, 30 patients (mean age 75.6 years, 90% with secondary MR, median LVEF 43%) with moderate-to-severe or severe symptomatic MR underwent treatment with the HighLife TMVR system [50]. Technical success was achieved in 90% of cases, with device success at 30 days in 83% [50]. At 1-year follow-up, no mitral valve reinterventions were required, and MR was eliminated or reduced to trace/mild in all successfully implanted patients [50]. Importantly, the device demonstrated a favorable hemodynamic profile (mean mitral gradient 5.1 mmHg) with no LVOT obstruction (mean gradient 2.0 mmHg), supporting its safety and feasibility in this high-risk cohort [50].

#### 3.3.6 Mitral Annular Calcification: A Persistent Challenge in TMVR

MAC is a chronic, degenerative process characterized by progressive calcium deposition at the fibrous base of the mitral valve. It is prevalent in the elderly, with reported rates up to 40% in this population, and is strongly associated with cardiovascular risk factors, including chronic kidney disease, hypertension, and coronary artery disease [51]. Beyond being a marker of systemic atherosclerosis, MAC contributes directly to mitral valve dysfunction, and when severe, poses significant challenges to both surgical and transcatheter mitral interventions [51].

Surgical management of MAC-associated mitral disease is complex due to difficulties in annular decalcification, increased procedural risk, and elevated early mortality in frail or comorbid patients [52]. Transcatheter options, including mitral valve-in-MAC procedures using balloon-expandable aortic valves, have shown improvements in symptoms and hemodynamics but are limited by high mortality rates and strict anatomic eligibility, since only a small subset of MAC patients has suitable circumferential annular calcification for anchoring [53].

**Fig. 1.**
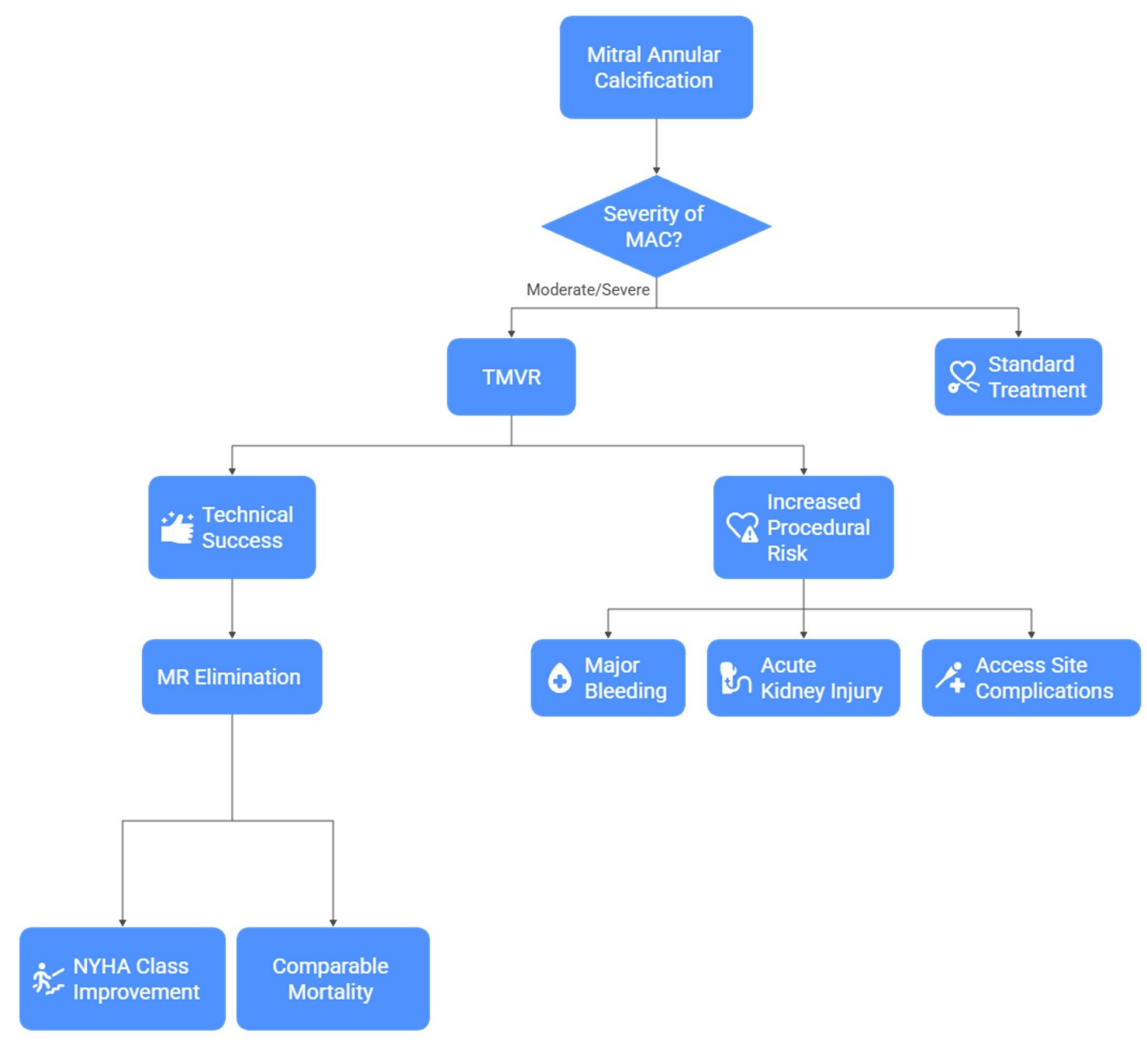
**Decision pathway for the management of mitral annular calcification (MAC)**. This schematic outlines the clinical decision process in patients with moderate or severe MAC. In appropriately selected patients, TMVR can achieve high technical success and effective elimination of mitral regurgitation, leading to improvements in NYHA functional class and mortality comparable to alternative therapies. However, TMVR is also associated with increased procedural risks, including major bleeding, acute kidney injury, and access-site complications. TMVR, transcatheter mitral valve replacement; MR, mitral regurgitation.

The advent of dedicated TMVR devices has opened new avenues for treating patients with severe MAC, many of whom are ineligible for MTEER due to poor leaflet quality or calcification in the grasping zone (Fig. 1).

In this context, the CHOICE-MI (Choice of Optimal Transcatheter Treatment for Mitral Insufficiency) registry offers critical insights into the feasibility of TMVR in patients with varying degrees of MAC [54]. In a recent analysis of 279 patients undergoing TMVR using eight dedicated devices, 57 patients (20.4%) had moderate or severe MAC. Despite higher baseline comorbidities, particularly extracardiac arteriopathy, patients with moderate/severe MAC had similar rates of technical success, MR elimination, and NYHA class improvement at 1 and 2 years compared to those with none or mild MAC [54]. Importantly, all-cause and cardiovascular mortality at 2 years were comparable between the groups [54]. However, the presence of moderate-to-severe MAC was associated with increased procedural risk, including higher rates of major bleeding (21.6% vs 8.8%), acute kidney injury (26.8% vs 8.2%), and access site–related complications [54]. These findings suggest that while MAC itself may not directly worsen long-term outcomes post-TMVR, it contributes to a more fragile procedural profile that is not currently captured by standard risk models. Given the rising prevalence of MAC and its overlap with aging and comorbidity, this study underscores the need for tailored transcatheter strategies, thorough preprocedural planning using advanced imaging, and ongoing device refinement. Recently, the SUMMIT-MAC trial showed that the Tendyne TMVR system can be safely and effectively implanted in high-risk patients with severe MAC, with high technical success and acceptable 30-day mortality [55]. At one year, most patients were free from death or heart failure hospitalization, and they experienced marked improvements in symptoms and quality of life [55]. Future randomized trials will be essential to establish evidence-based guidelines for this complex subset.

It should be noted that pre-procedural cardiac CT (CT) may assist in TMVR planning in patients with severe MAC. CT allows detailed assessment of the extent, distribution, and morphology of annular calcium, which determines the adequacy of the calcified landing zone for device anchoring and sealing [56]. Virtual valve implantation on CT is used to estimate the predicted neo-LVOT area, an important determinant of LVOT obstruction risk [56]. In addition to neo-LVOT measurements, CT helps evaluate the interaction between the prosthesis skirt and the calcified annulus, as sufficient circumferential contact between the valve skirt and calcium is required for anchoring and paravalvular leak prevention [56]. Implantation depth represents a key trade-off. Specifically, deeper ventricular positioning may improve sealing but increases the risk of LVOT obstruction [56]. However, a more atrial position may reduce LVOT compromise but predispose to paravalvular leak or device instability [56]. Furthermore, CT-based neo-LVOT prediction may be less accurate in Valve-in-MAC compared with valve-in-valve or valve-in-ring procedures because heavy calcification can alter the landing zone geometry and influence final device position [57].

Importantly, MAC does not uniformly represent a contraindication to TMVR. Rather, it should be considered a spectrum of anatomic risk. MAC may represent a relative contraindication when CT predicts severe LVOT obstruction, inadequate calcium for anchoring, or high embolization risk. Conversely, in selected patients, MAC may represent a modifiable procedural risk, particularly when CT planning demonstrates an adequate landing zone and when strategies to mitigate LVOT obstruction are feasible.

## 4. Patient Selection for Transcatheter Mitral Therapies

**Fig. 2.**
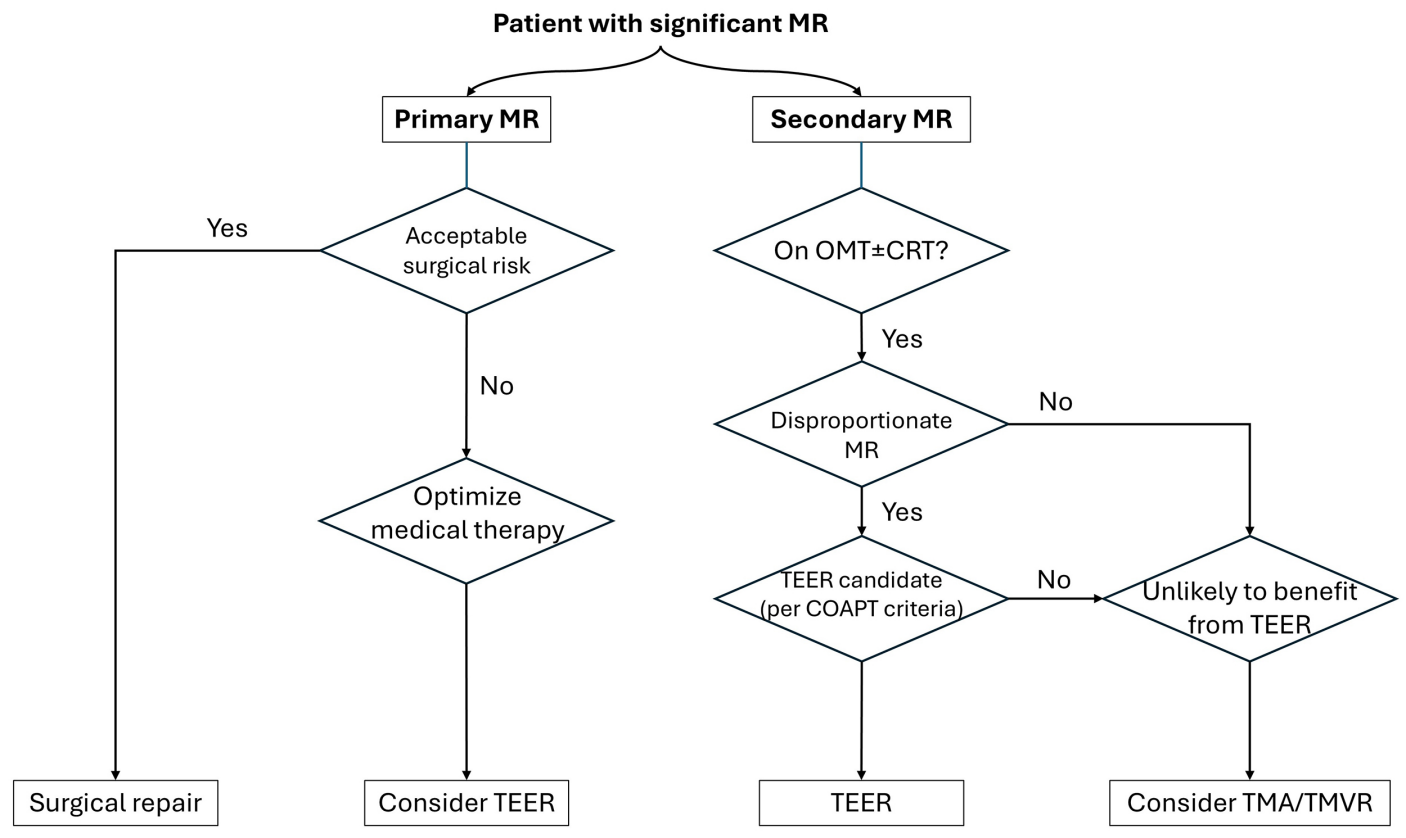
**Selection algorithm for transcatheter mitral therapies**. This flowchart outlines the decision-making pathway for patients with significant mitral regurgitation (MR), based on MR etiology (primary vs. secondary), surgical risk, medical therapy optimization, and anatomical considerations. MR, mitral regurgitation; MTEER, mitral transcatheter edge-to-edge repair; MAC, mitral annular calcification; OMT, optimal medical therapy; CRT, cardiac resynchronization therapy; TMA, transcatheter mitral annuloplasty; TMVR, transcatheter mitral valve replacement.

The decision-making process for selecting an appropriate transcatheter therapy in patients with significant MR is multifaceted and hinges on MR etiology, symptom burden, ventricular function, and procedural risk. Fig. 2 illustrates a simplified flowchart for therapy selection.

For patients with PMR, surgical repair remains the gold standard in those with acceptable operative risk. However, in high-risk or inoperable individuals, MTEER with devices such as MitraClip or PASCAL is a well-established alternative. Careful anatomical assessment is essential to confirm suitability for MTEER, including sufficient leaflet tissue, absence of prohibitive calcification, and favorable mitral valve geometry.

In contrast, SMR requires a nuanced approach. Initial management includes GDMT and, when indicated, CRT. In patients who remain symptomatic despite optimal medical therapy and demonstrate disproportionate MR relative to left ventricular dilatation (per COAPT criteria), MTEER has been shown to reduce hospitalization and mortality. Conversely, patients with proportionate MR or adverse valve anatomy may not derive benefit from MTEER and should be considered for alternative strategies such as transcatheter mitral annuloplasty or TMVR. Ultimately, all cases should undergo evaluation by a multidisciplinary heart team to ensure a tailored and evidence-based approach.

In patients with significant mitral annular calcification (MAC), treatment decisions require additional consideration, as extensive annular calcification may limit the feasibility of MTEER and influence the choice of TMVR. Careful pre-procedural CT assessment is therefore essential to evaluate anchoring stability and predict the risk of LVOT obstruction.

## 5. Clinical Implications, Limitations, and Future Directions

The rapid evolution of transcatheter therapies has substantially expanded treatment options for patients with significant mitral regurgitation who are unsuitable for surgery. From a clinical perspective, patient selection remains the most critical determinant of benefit. For secondary MR, optimization of guideline-directed medical therapy and device-based heart failure treatment remains the first step, with MTEER offering the most robust evidence of benefit in carefully selected patients with disproportionate MR and persistent symptoms. Annuloplasty-based approaches may provide an alternative strategy in selected patients with annular dilation and preserved leaflet mobility, while TMVR represents an important option for individuals with unfavorable anatomy for repair or recurrent MR after previous interventions. In clinical practice, these therapies should be considered complementary rather than competitive, with treatment decisions guided by detailed imaging assessment and multidisciplinary Heart Team evaluation.

Despite the promising results reported across transcatheter platforms, several limitations of the current evidence base should be acknowledged. First, most data for newer devices, particularly TMVR systems, derive from early feasibility studies, single-arm trials, or registries, often involving highly selected high-risk populations. Consequently, comparisons between different technologies remain indirect and susceptible to selection bias. Second, long-term durability and structural valve performance remain incompletely characterized, particularly for dedicated TMVR devices, where follow-up rarely exceeds one year. Third, uncertainties persist regarding optimal antithrombotic strategies and the incidence of valve thrombosis, an issue of particular relevance in transcatheter mitral prostheses due to their large prosthetic surface area and low-flow left atrial environment. Finally, procedural risk profiles, including LVOT obstruction, vascular complications, and bleeding, remain significant considerations that require careful preprocedural planning.

Future research should focus on several key areas. First, randomized trials comparing transcatheter strategies with optimized medical therapy or surgical intervention are needed to better define the role of newer technologies across different MR phenotypes. Second, studies evaluating head-to-head comparisons between repair and replacement strategies may help clarify the optimal treatment pathway for patients with complex mitral anatomy. Third, longer-term follow-up is essential to determine durability, valve thrombosis risk, and the need for reintervention after transcatheter mitral therapies. Advances in imaging-guided procedural planning, computational modeling, and device design are also likely to expand the anatomical eligibility for transcatheter interventions while reducing complications such as LVOT obstruction. Ultimately, continued integration of clinical trials, real-world registries, and device innovation will be essential to refine patient selection and establish evidence-based treatment algorithms for mitral regurgitation in the era of structural heart interventions.

## 6. Conclusion

Transcatheter interventions for MR have fundamentally transformed the management of both PMR and SMR in patients unsuitable for surgery. MTEER systems like MitraClip and PASCAL have demonstrated efficacy in well-selected patients, while annuloplasty and replacement technologies offer tailored alternatives for anatomically or clinically complex cases. As device iterations improve and randomized evidence accumulates, the landscape of MR therapy continues to shift toward a more individualized, anatomy-specific approach. Future studies comparing strategies head-to-head and clarifying optimal timing and patient selection will be key to maximizing outcomes in this evolving field.
